# *Citrobacter rodentium* possesses a functional type II secretion system necessary for successful host infection

**DOI:** 10.1080/19490976.2024.2308049

**Published:** 2024-02-01

**Authors:** Z Krekhno, SE Woodward, A Serapio-Palacios, J Peña-Díaz, KM Moon, LJ Foster, BB Finlay

**Affiliations:** a Department of Microbiology and Immunology, University of British Columbia, Vancouver, BC, Canada; b Michael Smith Laboratories, University of British Columbia, Vancouver, BC, Canada; c Department of Biochemistry and Molecular Biology, University of British Columbia, Vancouver, BC, Canada

**Keywords:** *Citrobacter rodentium*, type II secretion system, T2SS, mucin, attaching and effacing pathogen, mucosal invasion, mucus degradation, enteric infection, bacterial pathogenesis, host-pathogen interactions

## Abstract

Infectious diarrheal diseases are the third leading cause of mortality in young children, many of which are driven by Gram-negative bacterial pathogens. To establish successful host infections these pathogens employ a plethora of virulence factors necessary to compete with the resident microbiota, and evade and subvert the host defenses. The type II secretion system (T2SS) is one such conserved molecular machine that allows for the delivery of effector proteins into the extracellular milieu. To explore the role of the T2SS during natural host infection, we used *Citrobacter rodentium*, a murine enteric pathogen, as a model of human intestinal disease caused by pathogenic *Escherichia coli* such as Enteropathogenic and Enterohemorrhagic *E. coli* (EPEC and EHEC). In this study, we determined that the *C. rodentium* genome encodes one T2SS and 22 potential T2SS-secreted protein effectors, as predicted via sequence homology. We demonstrated that this system was functional *in vitro*, identifying a role in intestinal mucin degradation allowing for its utilization as a carbon source, and promoting *C. rodentium* attachment to a mucus-producing colon cell line. During host infection, loss of the T2SS or associated effectors led to a significant colonization defect and lack of systemic spread. In mice susceptible to lethal infection, T2SS-deficient *C. rodentium* was strongly attenuated, resulting in reduced morbidity and mortality in infected hosts. Together these data highlight the important role of the T2SS and its effector repertoire during *C. rodentium* pathogenesis, aiding in successful host mucosal colonization.

## Introduction

Diarrheal disease is the third most prominent cause of death in children under the age of five.^1^ Enteric pathogens, such as Enteropathogenic *Escherichia coli* (EPEC), are a leading driver of diarrhea-associated childhood mortality, particularly in developing countries.^2^ To establish an infection, these pathogens must create and maintain a replicative niche within the host gut using a plethora of virulence factors.^3^ One of the virulence factors associated with increased likelihood of symptomatic and lethal infections is a type II secretion system (T2SS).^4^ This system is widespread among Proteobacteria, and it is used to secrete fully folded proteins from the periplasm into the extracellular milieu or host tissue.^5^ In pathogens, it is associated with the secretion of a large number of sequence-unrelated and structurally dissimilar protein effectors, including lytic enzymes and toxins. These effectors are used to induce a variety of short- and long-range effects in the host environment across a wide range of processes, such as degradation of extracellular matrix, adherence to host surfaces, nutrient assimilation, and alterations in host ion flux. Therefore, T2SSs are increasingly recognized as important drivers of virulence.

The T2SS system itself spans the bacterial cell envelope and is classically composed of a complex of roughly 12–15 proteins.^6^ These structural proteins form three key components: the outer membrane complex with a secretin channel formed by GspD and GspS, the inner membrane complex or ‘assembly platform’ formed by GspCFLM and ATPase GspE, and a pseudopilus formed by GspGHIJK. In certain bacterial strains, GspD may be localized to the outer membrane with the help of GspA and GspB inner membrane peptidoglycan-interacting proteins. Interfering with key structural proteins, such as the GspE motor or GspK, which may control pseudopilus length, has been associated with loss of function of the T2SS.^6^

While the T2SS structure is conserved across a wide range of both pathogenic and nonpathogenic bacteria, it may be associated with different functions. In particular, nonpathogenic bacteria which harbor T2SSs tend to be free living, suggesting the importance of the system to environmental survival.^5^ Even within pathogens there is broad heterogeneity with regards to the full effector repertoires across bacterial species. Only one effector protein has been identified in EPEC, a metallopeptidase SslE, which has been proven vital for biofilm maturation and mucin degradation.^7,8^ Enterohemorrhagic *E. coli* (EHEC), a related infectious diarrheal disease agent, predominant in the developed world, has two characterized T2SS substrates, a mucinase StcE and YodA, whose enzymatic function is unknown, both of which are important for epithelial cell adhesion.^9,10^ However, it is common for bacteria to encode a large number of T2SS-associated effectors. Strikingly, proteomic analyses of secretomes of the plant pathogens *Burkholderia glumae* and *Burkholderia pseudomallei* revealed 34 and 48 proteins, respectively, as T2SS substrates with a wide range of function.^11,12^

EPEC and EHEC cause serious illness worldwide, mediated by their key shared virulence machinery, the type III secretion system (T3SS).^13^ The T3SS of EPEC and EHEC is well known to facilitate both intimate attachment to and the injection of protein effectors directly into host cells, and the mechanisms of action of many of these effectors has now been characterized.^14^ While T2SSs are becoming recognized as key virulence factors across pathogen species for the delivery of effector proteins, the full effector repertoire and function of the T2SS in EPEC and EHEC pathogenesis remains understudied. Unfortunately, the *in vivo* study of EPEC and EHEC is restricted by their poor ability to infect laboratory animals. Instead, pathogenesis research has exploited the natural murine pathogen *Citrobacter rodentium*, however, the T2SS in this bacterium has not been explored.^15^ *C. rodentium* acts as an excellent model of bacterial intestinal infection and it is the causative agent of transmissible murine colonic hyperplasia, a disease characterized by excessive proliferation of the colonic intestinal cells.^16^ Furthermore, all three pathogens cause a characteristic intestinal pathology in the form of attaching and effacing (AE) lesions, wherein they intimately attach to the apical plasma membrane of intestinal epithelial cells and effaces the microvilli brush border.^17^ Thus, *C. rodentium* serves as a valuable model for investigation of the role of the T2SS in the intestinal bacterial pathogenesis *in vivo*. In this study, we explored the function of *C. rodentium* T2SS under physiological and pathological conditions. Furthermore, we investigated the role of this system and some of its secreted effectors in mucin degradation, demonstrating a key role during colonization of mucosal surfaces.

## Materials and methods

### Bacterial strains and culture conditions

Bacterial strains, plasmids, and primers used in this study are listed in Tables S1–3 respectively. *C. rodentium* strain DBS100 was used in all assays. Unless otherwise stated, bacterial strains were maintained at −70°C and were streaked fresh from frozen onto lysogeny broth (LB) agar for colony growth overnight (18–20 hours) before inoculation into liquid LB broth overnight. This overnight liquid culture was then used in each assay.

### Effector prediction

*C. rodentium* strain ICC168 (identical to strain DBS100) proteome (UniProt accession number: UP000001889) was mined using the HMMER v3.2.1 against a curated database of known T2SS effectors.^18–21^ Proteins that passed default thresholds were further analyzed with STRING and common domains were identified with the Pfam database. STRING output was visualized using Cytoscape v3.9.1.^20,22–24^

### Generation of deletion mutants and complemented strains

Non-polar deletion of the *gspK, glpQ*, and *ROD_44811–41* genes in the *C. rodentium* strain DBS100 was carried out using the *sacB* gene-based allelic exchange method and the suicide vector pRE112.^25^ Briefly, upstream and downstream sequences of the target gene were amplified with Platinum SuperFI II DNA Polymerase (Thermo Fisher Scientific, Cat. No. 12361010), and combined with pRE112, linearized with SacI and KpnI, using Gibson Assembly.^26^ Deletion constructs were transformed into *E. coli* MC1061*λpir*, purified, and transformed into *E. coli* MFD*pir*. Suicide vector was then transferred into *C. rodentium* strain DBS100 through conjugation, where the vector integrated into the chromosome mediated by homologous recombination and double cross-over.^25^

Chromosomally complemented strain (Δ*gspK:gspK*) was generated by amplifying the *gspK* gene along with its native promoter. The construct was then cloned into the pUC18R6KT-mini-Tn7T plasmid system.^27^ Conjugation to the target *C. rodentium* strain was achieved via triparental mating using MFD*pir* as the donor and pTNS2 as the helper strain.

### Bacterial growth assays

Bacterial growth was assessed by culturing wild-type (WT) and indicated strains of *C. rodentium* in LB overnight before being spun down and resuspended in LB media to an optical density (OD) of 0.005. Bacterial growth was then monitored by measuring absorbance at 600 nm using a Biotek Synergy H1 plate reader over a period of 18 hours with reads every 10 minutes.

To determine the ability of WT and mutant *C. rodentium* to grow in the presence of mucin, bacteria were cultured overnight in LB as above, before inoculation to an OD of 0.005 into either mucin-rich media (Media recipe: 0.1 g/L peptone, 0.025 g/L yeast extract, 0.04 g/L sodium bicarbonate, 0.009 g/L glucose, 0.04 g/L type II porcine stomach mucin [Sigma-Aldrich], 1 µg/mL hemin, 0.01 g/L cysteine, 0.45 g/L K_2_HPO_4_, 0.45 g/L KH_2_PO_4_, 0.9 g/L NaCl, 0.9 g/L (NH_4_)_2_SO_4_, 0.09 g/L MgSO_4,_ 0.09 CaCl_2_) or media containing mucin as a sole carbon source (10X M9 salts supplemented with 1% type II porcine stomach mucin).^28,29^ Cultures were incubated for 3–24 hours at 37°C before dilution and plating for colony forming units (CFU) enumeration.

### Generation of plasmids for controlled expression of tagged putative T2SS effector proteins

*ROD_44811* and *ROD_44831* were amplified from *C. rodentium* strain DBS100 genomic DNA with Platinum SuperFI II DNA Polymerase (Thermo Fisher Scientific, Cat. No. 12361010) and cloned into pBAD33-trc-V5 plasmid (plasmid based on the pBAD33 backbone with an inserted multiple cloning site containing *trc* promoter and C-terminal sequence encoding the V5 tag in between the BamHI and XbaI restriction enzyme cut sites) using Gibson Assembly.^26,30^

In order to express *ROD_44811* in the chloramphenicol-resistant *C. rodentium* strains, the full gene, the *trc* promoter and the C-terminal sequence encoding the V5 tag were amplified from the pBAD33-trc-ROD_44811-V5 plasmid and cloned into a Zero Blunt TOPO plasmid (Thermo Fisher Scientific, Cat. No. 450245). Strains harboring this plasmid were used in the secretion assay in Figure S1.

### Protein secretion assays and immunoblotting

*C. rodentium* secreted proteins were analyzed as described previously with minor modifications.^31^ Briefly, bacterial cultures were incubated in LB at 37°C overnight, followed by static subculturing in Dulbecco’s Modified Eagle Medium (DMEM; Thermo Fisher Scientific, Cat. No. 11965092) supplemented with 1 mM Isopropyl ß-D-1-thiogalactopyranoside (IPTG; Thermo Fisher Scientific, Cat. No. 15529019) at 37°C in a 5% CO_2_ atmosphere for 6 hours. Samples were collected after 6 hours and subjected to centrifugation at 3,000 × *g* for 20 min in 30 mm glass tubes. Bacterial pellets were saved when convenient, and 2.7 mL of each supernatant was separated into fresh tubes and 300 μL of 100% trichloroacetic acid (TCA; Sigma-Aldrich Cat. No. T6399) and 10 μg bovine serum albumin (BSA) was added per tube. Proteins were allowed to precipitate at 4°C overnight. Subsequently, the proteins were concentrated by centrifugation at 13,000 × *g* for 30 min and resuspended in 30 μL of 1× Laemmli loading buffer, boiled for 10 min, and resolved by 11% SDS-polyacrylamide gel electrophoresis (PAGE) and, subsequently, transferred to 0.2 μm pore size nitrocellulose membrane (Sigma-Aldrich; Cat. No. GE10600001) using a wet transfer chamber.

The membrane was blocked in 5% BSA and incubated with 1:5,000 dilution of mouse monoclonal anti-V5 antibody (Thermo Fisher Scientific, Cat. No. R960–25). It was then washed with 1× tris-buffered saline (TBS) containing 0.05% Tween 20 and incubated with a 1:10,000 dilution of secondary anti-mouse antibodies coupled to Dye-IR800 fluorophores (Azure Biosystems, Cat. No. AC2135) and imaged with Azure Sapphire Biomolecular Imager (Azure Biosystems, Dublin, CA, United States). To confirm equal loading, the membrane was stained with Ponceau S solution (Sigma-Aldrich, Cat. No. P7170-1 L).

### Cell culture and epithelial cell attachment assays

The human colonic epithelial cell line HT-29-MTX (European Collection of Authenticated Cell Cultures, Cat. No. 12040401) was cultured in DMEM supplemented with 10% v/v heat-inactivated fetal bovine serum (FBS; Gibco, Cat. No. A3160402), 1% v/v Glutamax (Gibco, Cat. No. 35050061), 1% v/v non-essential amino acids (NEAA; Gibco, Cat. No. 11140–050) and 100 U/mL PenStrep (Gibco, Cat. No. 15140122) and maintained at 37°C in a 5% CO_2_ atmosphere.^32^ Cells were subjected to at least 4 routine passages based on confluency before use in subsequent assays.

For use in infection assays HT-29-MTX cells were seeded at a density of 120,000 cells/mL and allowed to differentiate for 21 days to induce robust mucus production as described.^33,34^ Culture media was changed every 2–3 days. Competitive index (CI) assays were carried out following inoculation at multiplicity of infection (MOI) 100 of equal parts WT CmR and either WT or effector mutant strains (assays in Figure S5) or equal parts of WT *C. rodentium* and chloramphenicol resistant WT, Δ*gspK*, or Δ*gspK:gspK* (assays in Figure 5(d)). Inoculation was followed by centrifugation for 5 min at 1000 rpm to synchronize the infection. After 4 hours, cells were washed 5 times with phosphate-buffered saline (PBS) to remove unattached bacteria. Cells were then detached by incubation in 0.01% Triton X-100 followed by serial dilution and plating on agar plates with or without chloramphenicol (30 µg/mL) to allow for enumeration of both inoculated strains. CI was calculated as the proportion of CmR bacterial cells attached to HT-29-MTX cells compared to the proportion of CmR cells in the inoculum.

### RNA extraction and RT-qPCR

WT *C. rodentium* was grown in LB overnight, and sub-cultured into LB, DMEM, or spent DMEM tissue culture media (supplemented with 10% v/v FBS, 1% v/v NEAA, and 1% v/v Glutamax, and collected after 2 days of culture of HT-29-MTX cells) at 1:30 dilution and grown for a further 3 hours at 37°C in a 5% CO_2_ atmosphere. Bacterial RNA was stabilized with RNAprotect Bacteria Reagent (Qiagen, Cat. No. 76506) and stored at −70°C before extraction using a RNeasy Mini Kit (Qiagen, Cat. No. 74104). cDNA was synthesized using a QuantiTect Reverse Transcription Kit (Qiagen, Cat. No. 205313). Quantitative RT-PCR was performed using QuantiNova SYBR Green RT-PCR Kit (Qiagen, Cat. No. 208056) and various primer pairs (see Table S3). *dnaQ* was used as the endogenous control.

### Animal infections and ethics

Animal experiments were performed in accordance with the guidelines of the Canadian Council on Animal Care and the University of British Columbia (UBC) Animal Care Committee according to Animal Care Protocol A20–0187. All mice used in this study were obtained from Jackson Laboratory (Bar Harbor, ME) and allowed to acclimatize to the facility for a period of one week upon arrival before infection. Mice were maintained in a specific pathogen-free facility at UBC on a 12-hour light-dark cycle.

All mice were gavaged orally with 10^8^ CFU of WT or mutant *C. rodentium* DBS100 (100 µL volume) after which mice were monitored daily throughout the 7-day infection period for weight loss and clinical symptoms. 7-week-old female C57BL/6J mice were used in experiments to determine *C. rodentium* burden. Mice were euthanized at experimental endpoint by isoflurane anesthesia followed by carbon dioxide inhalation. To determine changes to morbidity and mortality in response to WT or mutant *C. rodentium*, 7-week-old female C3H/HeJ mice were used, which are highly susceptible to infection. These mice were infected as described above and were monitored twice daily for weight loss and clinical symptoms and euthanized upon reaching humane endpoint.

### *In vivo* sample collection and processing

Fecal samples were collected from infected mice every two days post-infection (p.i.) in order to enumerate *C. rodentium* shedding. Fecal samples were combined with 1 mL of PBS and homogenized in a Mixer Mill MM 400 (Retsch) for 5 minutes at 25 1/s before dilution and plating on MacConkey agar. Plates were incubated at 37°C for 18–20 hours before counting resultant bacterial colonies.

At experimental endpoint, the cecum and colon of the gastrointestinal tract were collected to determine intestinal colonization, and the spleen was collected in order to determine systemic spread. All tissue samples were collected in 1 mL PBS and homogenized using a FastPrep-24 (MP Biomedicals) at 5.5 m/s for 2 min. Homogenized samples were then further diluted in PBS for plating on MacConkey agar. After 18–20 hours of growth at 37°C bacterial colonies were counted to enumerate CFU.

In order to determine mucosal and lumenal colonization of *C. rodentium*, a subset of cecum and colon samples were opened longitudinally and gently scraped to collect lumenal content. The remaining tissue was washed twice in PBS before collection to represent the mucosal population. Samples were then processed as described above.

### Histological analysis

Distal colon samples (0.5 cm) were fixed in 10% formalin for 24 h and then submersed in 70% ethanol for long term storage. Fixed tissues were processed, paraffin embedded, and sectioned at 5 μm by the UBC Diagnostic and Research Histology Laboratory (Vancouver, BC, Canada). Paraffin-embedded sections (FFPE) were then either stained with hematoxylin and eosin (H&E) using standard techniques or treated with sodium citrate antigen de-masking solution prior to immunofluorescence staining. Primary antibodies were used at 1:200 dilution for rabbit anti-Ki67 (Thermo Fisher Scientific, Cat. No. RM-9106-S1) and at 1:500 dilution for rat anti-CDH1 (Thermo Fischer Scientific, Cat. No. 13–1900), followed by secondary antibodies used at a 1:2000 dilution (goat anti-rabbit, Alexa Fluor 488 [Thermo Fisher Scientific, Cat. No. A-11034], goat anti-rat, Alexa Fluor 568 [Thermo Fisher Scientific, Cat. No. A-11077]) and nuclear counterstains (DAPI [Thermo Fisher Scientific, Cat. No. D1306]). H&E-stained sections were analyzed using a 20× lens objective on the Zeiss Axioskop 2 MOT microscope. At least 30 crypts from each section from each individual mouse were measured, and an average crypt length for each mouse was calculated using QuPath v0.3.2.^35^ Ki67-stained slides were imaged with a Zeiss Axio Imager M2 microscope and computer-processed using Zen imaging software (Carl Zeiss MicroImaging GmbH, Germany). CellProfiler v4.2.1 was used to automatically detect all nuclei and Ki67-positive nuclei in epithelial (CDH1-positive) cells.^36^

### 16S rRNA amplicon sequencing

Fecal pellets were freshly collected and stored at − 70°C until analysis. The microbial DNA was extracted using QIAGEN PowerFecal kits, and the V4 variable region of the 16S rRNA gene was amplified using 515F 806 R primers (Table S3) indexed with unique identifiers. Indexed PCR products were pooled and sequenced on an Illumina MiSeq platform using V2 technology.^37^ Raw reads were quality-filtered and processed using DADA2 implemented in QIIME2.^38,39^ Taxonomy was assigned using a Naive Bayes classifier trained on the SILVA 138 database of the 515/806 region at 100% amplicon sequence variants (ASV) cutoffs.^40^ Further filtering was performed in R using the phyloseq package to remove singletons and rarefy samples to a uniform sequencing depth of 10,500 reads.^41,42^ Beta diversity analyses were performed using phyloseq in R, and the results were plotted using ggplot2. Differential abundance analysis was conducted on unrarefied samples using DESeq2 (version 1.34.0).^43^

### Cytokine measurements

Fresh cecum samples were collected at 7 days post-infection into 1 mL of PBS containing a complete EDTA-free protease inhibitor cocktail (Roche Diagnostics). Tissues were then homogenized using a FastPrep-24 (MP Biomedicals) at 5.5 m/s for 2 min, followed by centrifugation at 16,000 × *g* for 20 min at 4°C, and collection of resultant supernatant. A mouse inflammation cytometric bead array (CBA) kit (BD Biosciences, Cat. No. 552364) was used to measure the concentration of cytokines within supernatant samples according to the manufacturer’s instructions. Measurements were made on an Attune NxT flow cytometer (Thermo Fisher Scientific) and cytokine concentrations were normalized to tissue weight.

### Statistical analysis

Statistical analysis was performed in GraphPad Prism v.9.4.1 (www.graphpad.com) and R v.4.2.2.^41^ All statistical tests used are described in the figure legends. Unless otherwise stated, analysis of non-normally distributed data was performed using Mann-Whitney tests to compare two groups and Friedman tests for more than two groups with Dunn’s multiple comparisons test. Aggregate results represent the mean ± standard error, and statistical significance is represented by *p < .05, **p < .01, ***p < .001 and ****p < .0001.

## Results

### *C. rodentium* possesses a functional type II secretion system

**Figure 1.**
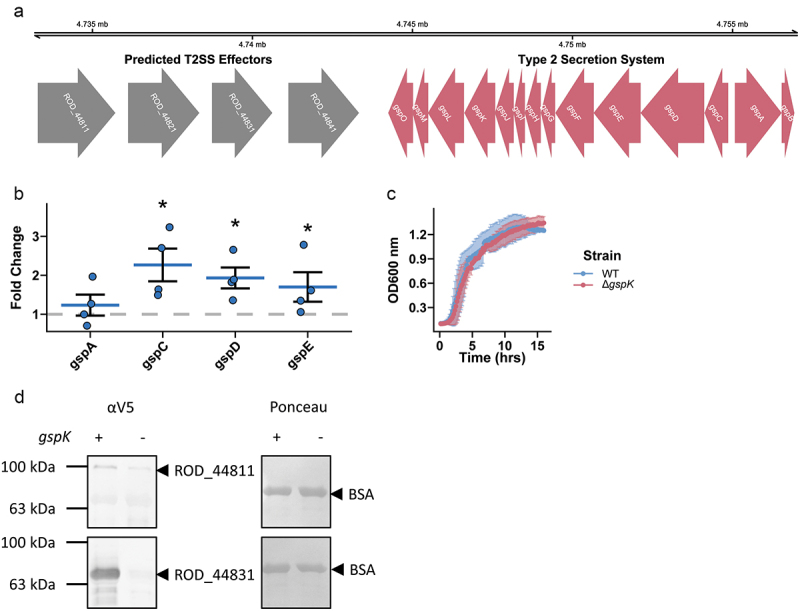
A type 2 secretion system is present and functional in *C. rodentium*. (a) A complete T2SS operon is present in the *C. rodentium* genome and 4 predicted effector proteins are located in close proximity to the operon (colored red and grey respectively). (b) Selected T2SS gene expression changes calculated in *C. rodentium* cultured in DMEM for 3 hours using a ΔΔCt method with *dnaQ* and *C. rodentium* grown in LB media used as reference. *n* = 4, Wilcoxon signed-rank test, error bars are standard error of the mean. (c) Growth dynamics of wild-type (WT) or T2SS knock-out (Δ*gspK*) strain in LB measured by optical density of the culture. *n* = 3, error bars are standard deviation. (d) Protein secretion assay showing secretion of putative effector proteins in the WT and ∆*gspK* strains. Western blot detection of C-terminally V5-tagged ROD_44811 and ROD_44831 proteins expressed ectopically in WT and ∆*gspK* strains. Ponceau staining for BSA shown to demonstrate equal loading. *n* = 3 independent experiments, representative blots shown.

*C. rodentium* encodes a complete T2SS in its genome (Figure 1(a)).^44^ Petty *et al*. ^18^ discovered nine putative substrates, four of which are positioned closely upstream of the T2SS locus, suggesting possible roles as T2SS-associated secreted effector proteins. By gene expression analysis we found that gut mimicking conditions in DMEM media stimulated expression of the structural genes *gspC, gspD*, and *gspE*, suggesting active transcription of T2SS-associated genes (Figure 1(b)). As the T2SS of other bacterial pathogens has previously been shown to be important in nutrient acquisition, we sought to determine how the loss of T2SS activity would affect *C. rodentium* growth dynamics *in vitro*. This was done by deleting the *gspK* gene, which encodes a structural component of the T2SS necessary for its formation.^45^ We found that the loss of T2SS did not affect *C. rodentium* growth dynamics *in vitro* in conventional laboratory media (Figure 1(c)).^5^ However, we did find that secretion of two T2SS-associated effectors was reduced in the ∆*gspK* strain as compared to WT, indicating that knocking out GspK does indeed impair the function of the *C. rodentium* T2SS (Figure 1(d)). Importantly, in-chromosome complementation of the *gspK* gene restored T2SS-associated secretion (Figure S1). Taken together, these data suggest that *C. rodentium* does possess a functional T2SS that can be manipulated through deletion of the key structural gene *gspK*.

### Loss of the T2SS results in reduced pathogen colonization, intestinal tissue pathology, and systemic inflammation in vivo

**Figure 2.**
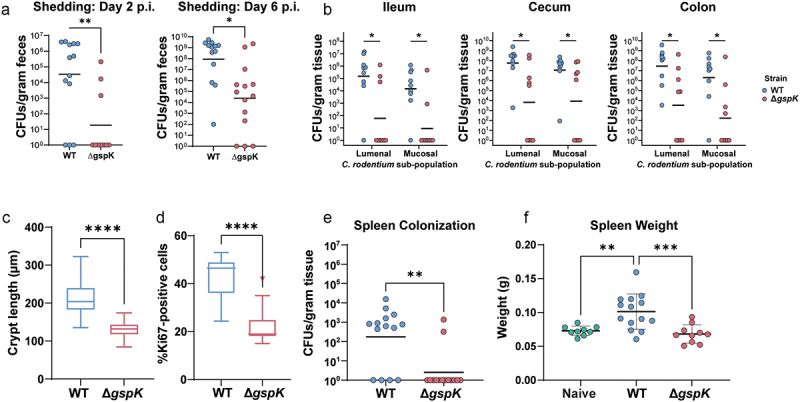
*C. rodentium* T2SS is necessary for successful infection in C57BL/6J mice. (a) *C. rodentium* shedding was assessed by CFU enumeration from feces shed daily post- infection and normalized to fecal weight. (b) Mucosal and lumenal sub-populations of *C. rodentium* in the cecum, ileum, and colon were measured by plating either the lumenal contents or the mucosal tissues on MacConkey agar and counting colony-forming units (CFU). At least *n* = 5 mice per group, Wilcoxon signed-rank test. (c,d) Hyperplasia was determined by either measuring the crypt length in (C) hematoxylin and eosin (H&E) stained murine intestinal samples or (D) by calculating the proportion of Ki67-positive epithelial (CDH1-positive) cells (as determined by immunofluorescent staining). There were at least *n* = 5 mice per group and a minimum of 30 crypts were measured per H&E-stained tissue section. (e,f) Systemic spread was assessed by measuring spleen colonization (E) and spleen weight (splenomegaly) (F). * - *p* < .05, ** - *p* < .01, *** - *p* < .001, **** - *p* < .0001. Wilcoxon signed-rank test.

To elucidate the role of the *C. rodentium* T2SS within its natural host, we performed an infection in C57BL/6J mice. In these mice, *C. rodentium* causes self-limiting colitis, which typically resolves within three weeks post-infection.^46^ We found that mice infected with the ∆*gspK* strain exhibited reduced pathogen shedding in the feces over the course of infection, indicating a possible colonization defect (Figure 2(a)). To further investigate intestinal colonization, mice were euthanized at peak infection (7 days post-infection [p.i.]). Loss of T2SS activity resulted in decreased lumenal and mucosa-associated pathogen burden, demonstrating a generalized defect in colonization across gastrointestinal regions (Figure 2(b)). We next sought to determine whether intestinal pathology was consistent with decreased pathogen burden. Indeed, the Δ*gspK* strain caused significantly less hyperplasia of the intestinal epithelium, a hallmark feature of *C. rodentium* infection, as evidenced by both reduced crypt length and proportion of Ki67-positive, or actively proliferating, cells (Figure 2(d); representative images in Figure S2A,B).^47^ By investigating local intestinal inflammation, we further found a decrease in cecal levels of the inflammatory cytokines MCP-1 and IL-6 (Figure S2C). Furthermore, infection with the ∆*gspK* strain resulted in decreased systemic spread at peak infection, as indicated by the absence of detectable spleen colonization in the majority of infected mice, as well as a lack of characteristic spleen enlargement as a result of the systemic inflammatory response to *C. rodentium* (Figure 2(e,f)). Overall, these data suggest that a functional T2SS is necessary for host infection by *C. rodentium* and associated tissue pathology.

### A functional type II secretion system is required for lethality within a susceptible host

**Figure 3.**
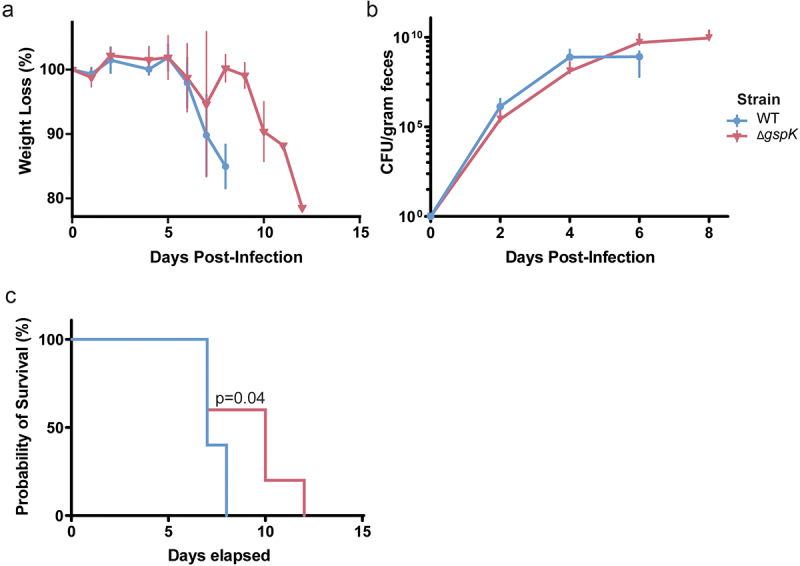
*C. rodentium* T2SS is necessary for successful infection in C3H/HeJ mice. (a) Weight loss post-infection in WT and T2SS-deficient strains, presented as a proportion of weight (g) measured before *C. rodentium* infection. *n* = 5 for each group, error bars indicate standard deviation. (b) Bacterial load of *C. rodentium* shed in the feces post-infection as determined by selective plating and counting CFU. *n* = 5 for each group, error bars indicate standard deviation. (c) Probability of survival represents the proportion of mice that have not reached the pre-determined humane endpoints with regards to infection symptoms. *n* = 5 for each group. Probability of survival in WT- and Δ*gspK*-infected mice was compared using the log rank test.

In order to determine how this decreased mucosal colonization throughout the gut would manifest in a more susceptible host, the effect of the T2SS on lethality was assessed in C3H/HeJ mice which are known to succumb to *C. rodentium* infection within 6–10 days.^48^ Given the importance of the T2SS for bacterial pathogenesis in other organisms, we hypothesized that the T2SS and its secreted effectors would similarly play an important role during *C. rodentium* infection.^5^ Mice infected with the WT or Δ*gspK* strain exhibited similar patterns of weight loss over the course of infection (Figure 3(a)), though significant losses were delayed by a period of roughly 4 days in mice infected with T2SS-deficient *C. rodentium*. Likewise, we found that loss of the T2SS did not significantly influence the fecal bacterial burden, suggesting similar levels of intestinal colonization (Figure 3(b)). Nevertheless, lack of a functional T2SS resulted in profoundly longer mouse survival (Figure 3(c)) of up to 4 days, indicating a substantial attenuation in the absence of the T2SS. Taken together, it is clear that the *C. rodentium* T2SS is associated with lethal infection of a susceptible host.

### The *C. rodentium* T2SS has roles in mucin degradation and utilization

**Figure 4.**
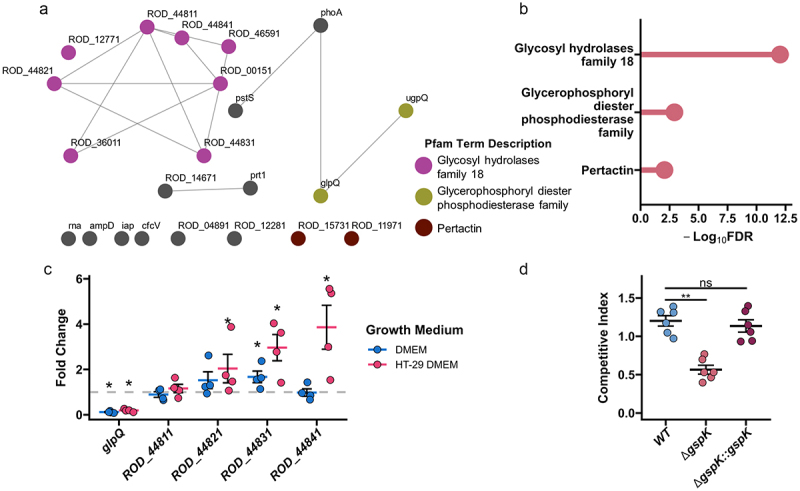
C. rodentium T2SS enables mucin degradation. (a) A network of association of the predicted T2SS effectors. A node-to-node connection indicates that the strength of association has passed a threshold confidence of 0.4 based on STRING analysis. Nodes are coloured based on the gene belonging to one of the statistically enriched pfam protein domains: magenta for “glycosyl hydrolases family 18”, olive for “glycerophosphoryl diester phosphodiesterase family”, brown for “pertactin”, and grey for not being enriched. (b) Lollipop plot showing significantly enriched domains among the predicted effectors. FDR is false discovery rate. (c) Selected T2SS gene expression changes calculated in *C. rodentium* cultured for 3 hours in either fresh DMEM or DMEM obtained following 2 days of HT-29-MTX culture using *dnaQ* and *C. rodentium* grown in LB media as reference. *n* = 4, Wilcoxon signed-rank test, error bars represent standard error of the mean. (d) Competitive index (CI) calculated via co-infection of intestinal epithelial cell line HT-29-MTX with either chloramphenicol-resistant isogenic WT, Δ*gspK*, or Δ*gspK:gspK C. rodentium* strains and WT *C. rodentium*. CI is indicative of the proportion of adherent bacteria belonging to each strain following a 4-hour infection period compared to the corresponding ratio of strains in the inoculating population. Kruskal-Wallis test followed by Dunn’s multiple comparisons test. *n* = 6.

Having demonstrated the importance of the T2SS to host intestinal colonization, we next sought to determine the specific function of the system *in vivo*. To do this, we used predictive tools to identify putative protein effectors. We first searched the *C. rodentium* proteome against BastionHub, a curated database of T2SS substrates, which identified 22 potential effector proteins, including seven of the nine previously reported (Table 1).^19,20^ Analysis of these effectors with STRING further indicated tight associations between some effectors (Figure 4(a)).^22^ Interestingly, almost a third of the proteins possessed glycosyl hydrolase family 18 (GH18) domains (Figure 4(b)).^49^ GH18 proteins can be both catalytically active chitinases and non-hydrolytic proteins that function as carbohydrate-binding molecules.^50^ These enzymes have been shown to interact with a variety of substrates, such as mucin, chitin, and peptidoglycan.^50–52^

**Table 1.** Putative T2SS substrates in *C. rodentium*.

Gene names/IDs	Uniprot ID	Description	E-value
*ROD_44811*	D2TMG2	Putative polysaccharide degrading enzyme	1.20E–292
*phoA*	D2TKW7	Alkaline phosphatase	4.30E–221
*glpQ*	D2TGZ0	GP-PDE domain-containing protein	2.20E–46
*ROD_00151*	D2TGJ6	Glyco_18 domain-containing protein	3.40E–40
*ROD_36011*	D2TRQ1	Glyco_18 domain-containing protein	2.80E–30
*ROD_44821*	D2TMG3	Glyco_18 domain-containing protein	4.90E–30
*ROD_46591*	D2TPC1	Glyco_18 domain-containing protein	1.80E–29
*ROD_44831*	D2TMG4	Glyco_18 domain-containing protein	6.10E–29
*ROD_44841*	D2TMG5	Glyco_18 domain-containing protein	1.90E–28
*prt1*	D2TTD5	Neutral metalloproteinase	1.40E–25
*rna*	D2TMQ6	Ribonuclease I	2.80E–23
*ROD_12771*	D2TUI8	Glyco_18 domain-containing protein	2.30E–18
*ROD_04891*	D2TLR0	Putative exported protein	1.30E–10
*pstS*	D2THB1	Phosphate-binding protein PstS	1.30E–06
*ugpQ*	D2TLJ5	GP-PDE domain-containing protein	1.10E–05
*iap*	D2TKL3	Peptidase_M28 domain-containing protein	1.60E–05
*ROD_12281*	D2TUD8	Uncharacterized protein	1.80E–05
*ROD_11971*	D2TTW8	Autotransporter domain-containing protein	7.80E–05
*ampD*	D2TGP5	N-acetylmuramoyl-L-alanine amidase domain-containing protein	1.00E–04
*cfcV*	D2TPB9	Peptidase_M23 domain-containing protein	0.00011
*ROD_14671*	D2TIA5	Serine protease	0.00013
*ROD_15731*	D2TJC3	Autotransporter domain-containing protein	0.00021

Given the large proportion of the T2SS substrates predicted to be involved in polysaccharide degradation, we investigated the effects of one of the most frequently encountered intestinal carbohydrates, mucin, on the expression of the structural T2SS genes and some of the predicted effectors. We chose to focus on the four effectors within the *ROD_44841–11* locus (*ROD_44841, ROD_44831, ROD_44821, ROD_44811*), as their close genomic proximity to the T2SS locus suggested them as most likely substrates (Figure 1(a)). We further chose to investigate the role of GlpQ, as a representative of the “glycerophosphoryl diester phosphodiesterase family” of predicted effectors. In order to investigate these proteins as type II secreted effectors, we grew *C. rodentium* in either LB media, DMEM, which is conventionally used to induce expression of virulence genes in AE pathogens, or DMEM after two days of culturing with mucus-producing intestinal cell line HT-29-MTX, rich in mammalian cell metabolites and mucin (referred to as HT-29 DMEM from here on).^32,53,54^ Growth in unprocessed DMEM resulted in a significant upregulation of genes encoding the structural T2SS components *gspC*, *gspD*, and *gspE*, but had no effect on the adjacent GH18-containing effectors, with the exception of a significant increase in expression of *ROD_44831* (Figures 1(b) and 4(c)). Intriguingly, growth in HT-29 DMEM caused a significant upregulation of transcript levels of the *ROD_44841, ROD_44831*, and *ROD_44821* genes. Furthermore, we noted decreasing strength of induction further from the start of the *ROD_44841–11* locus. Surprisingly, transcript expression of predicted effector GlpQ was reduced in both DMEM and HT-29 DMEM. This effector contains a “glycerophosphoryl diester phosphodiesterase” domain, and thus, is unlikely to be regulated by mucin, though lack of induction by pure DMEM suggests a mechanism of regulation that is distinct from that of the *ROD_44841–11* locus. These data suggest that transcript level regulation of select T2SS-associated effectors is moderated by the presence of host metabolites, including mucin.

Considering the importance of the mucus layer as a key niche of *C. rodentium* within the colon, we hypothesized that the ability to degrade mucin would allow not only for intestinal colonization but also provide a key nutrient source for invading bacteria. We therefore sought to investigate the potential of the T2SS to facilitate the degradation of mucin specifically. To do this, we grew WT and Δ*gspK C. rodentium* in media containing porcine stomach mucin. Indeed, we found that T2SS-deficient *C. rodentium* was unable to grow as efficiently as the wild-type strain in mucin-rich media (Figure S3A). A similar trend was noted following culture in a medium containing M9 salts and 1% mucin, though both strains were unable to grow as efficiently using mucin as a sole carbon source (Figure S3B). Therefore, it is clear that a functional T2SS plays a role in the degradation and utilization of mucin as a carbon source.

While nutrient acquisition from the mucus layer is important for host intestinal colonization, mucin degradation is also important to allow invading *C. rodentium* access to the host epithelium for intimate attachment and secretion of virulence factors into host cells.^55^ To investigate the importance of the T2SS for mucus layer penetration we infected a mucus-producing intestinal epithelial cell line HT-29-MTX with both wildtype and ∆*gspK C. rodentium* strains. The ∆*gspK* mutant was significantly impaired in attachment to HT-29-MTX cells in competition with the wildtype strain, and normal phenotype was restored upon complementation of the *gpsK* gene (Figure 4(d)). Interestingly, deletion of *gspK* in the context of infection in C57BL/6J mice led to inability of *C. rodentium* to displace the resident microbiota (Figure S4A). One of the bacteria that was not displaced was *Akkermansia muciniphila*, a prevalent mucin-degrading microbe that resides in the mucus layer near intestinal epithelial cells (Figure S4B).^56,57^ This further suggests that a functional T2SS is required for successful penetration and degradation of the mucus layer *in vivo*.

To determine whether a lack of predicted mucin-degrading effector proteins would similarly result in impaired mucus layer colonization, two mutant strains were created: one lacking the *ROD_44841–11* locus (∆*ROD_44841–11*) as well as a mutant in *glpQ* (∆*glpQ*). By using these strains to infect HT-29-MTX cells, we did not observe a defect in attachment with the ∆*ROD_44841–11* strain when in competition with WT *C. rodentium* (Figure S5A). This suggests that other type II secreted effectors may contribute to mucus degradation. Similarly, the ∆*glpQ* mutant strain did not display a decreased ability to degrade the mucin layer and attach to the HT-29-MTX cells (Figure S5A). This phenotype was consistent with our previous observation that *glpQ* expression was not activated in the presence of mucin (Figure 4(c)). Importantly, neither effector deletion affected growth dynamics in LB, and so these genes were unlikely to be required for general bacterial fitness (Figure S5B). Taken together, these data support the function of the *C. rodentium* T2SS in the degradation of mucin, which aids in both nutrient acquisition and epithelial colonization.

### Deletion of mucus-degrading type II secreted effectors results in colonization defects

**Figure 5.**
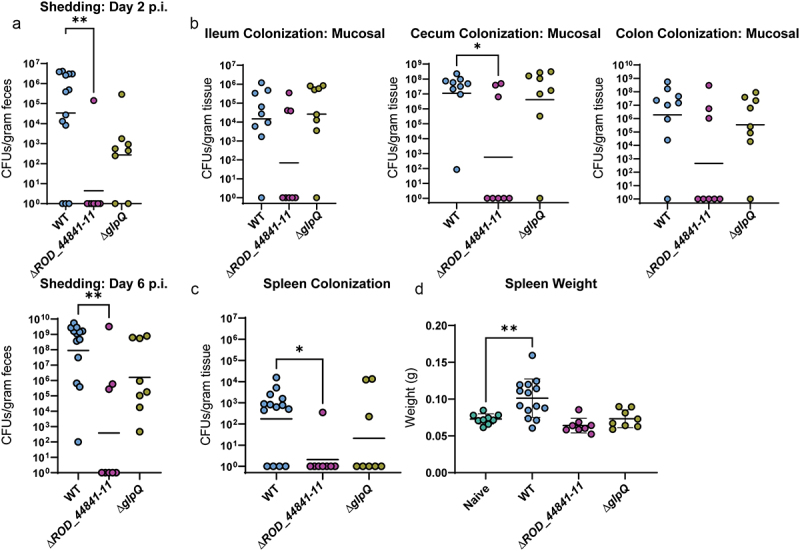
Individual T2SS effectors aid successful infection in C57BL/6J mice. WT-infected mice in this panel are the same mice as WT-infected mice in Figure 3. (a) *C. rodentium* shedding was assessed by CFU enumeration from feces and normalized to fecal weight. Kruskal-Wallis test followed by Dunn’s multiple comparisons. (b) Mucosal sub-populations of *C. rodentium* in cecum, ileum, and colon were measured by plating the mucosal tissue on MacConkey agar and counting CFU. Kruskal-Wallis test followed by Dunn’s multiple comparisons. (c) Systemic spread was assessed by measuring spleen weight (splenomegaly) and spleen colonization. * - *p* < .05, ** - *p* < .01. These comparisons were assessed with ANOVA and Dunnett’s multiple comparisons test.

Considering the striking decrease in colonization as a result of interfering with the structural components of the T2SS and thereby a full deletion of the system, we next sought to determine whether the deletion of predicted effector proteins would have similar effects on murine infection. To do this, we infected C57BL/6J mice with the Δ*ROD_44841–11* and the Δ*glpQ C. rodentium* mutants. As we expected, deletion of the *ROD_44841–11* locus led to a strong defect in infection dynamics evident as early as 2 days post-infection (Figure 5(a)). Intriguingly, Δ*glpQ C. rodentium* also had a colonization defect, although not as pronounced as the strains lacking the whole T2SS or the mucin-degrading effectors. Again, to further investigate the role of these effectors in infection, mice were euthanized at peak infection. The Δ*ROD_44841–11* mutant showed a strong decrease in gastro-intestinal colonization, both in the lumenal and mucosa-associated bacterial levels, especially in the cecum and colon at 7 days p.i., while the Δ*glpQ* strain had colonization levels equivalent to that of WT *C. rodentium* (Figure 5(b) and S6). Furthermore, deletion of either *ROD_44841–11* or *glpQ* strongly impaired *C. rodentium* mutant strains ability to cause systemic infection, as evidenced by the lack of spleen colonization or spleen enlargement, compared to WT *C. rodentium* (Figure 5(c,d)). Taken together, these data suggest that the *ROD_44841–11* locus and, to a lesser extent, *glpQ* are important for maintaining proper infection dynamics, host colonization, and associated tissue pathology.

## Discussion

Pathogens employ sophisticated strategies to overcome barriers imposed by the host environment and establish successful infection. One such strategy involves the use of a T2SS, which allows for the secretion of proteins into the extracellular space. This system is conserved across a wide array of bacteria within the phylum Proteobacteria, including intestinal pathogens *Salmonella enterica*, *Vibrio cholerae*, and *Yersinia enterocolitica*.^5^ Notably, it is also present in AE pathogens, such as EPEC and EHEC, and its presence is associated with higher morbidity and mortality as a result of EPEC infection.^4^ In fact, this system has been shown to play a major role in EPEC and EHEC infections *in vitro* due to its importance for epithelial cell adhesion and intestinal colonization.^7,10^ *C. rodentium*, a natural murine pathogen which shares key virulence strategies with EPEC and EHEC, also possesses a T2SS, thus facilitating further investigation of the T2SS during AE pathogenesis *in vivo*.^16,18^ However, despite the opportunity to investigate the T2SS function within its natural host, the system still remains largely underexplored. In this study, we not only confirm the functionality of the T2SS possessed by *C. rodentium* but highlight its importance to host colonization success.

We clearly demonstrate the necessity of a functional T2SS for stable intestinal colonization across the large intestine and further propose a particular importance during early colonization events, as demonstrated by the early disparity in fecal shedding of WT and T2SS-deficient strains as early as day 2 post-infection (Figure 2(a)). This phenotype could arise due to the inability of the mutant strain to compete with resident microbiota, an established early barrier to epithelial colonization (Figure S4).^58^ One of the common ways for intestinal pathogens to establish a niche during infection is to have expanded metabolic preferences, allowing them to outcompete and ultimately displace niche-resident commensal microbes.^59^ As we found that almost a third of predicted *C. rodentium* T2SS substrates contain a GH18-domain (chitinase or endo-β-N-acetylglucosaminidase domain), it is likely that carbohydrate degradation is an important role for this system, aiding in nutrient acquisition within the gut environment and allowing for enhanced pathogen growth (Figure 4(a,b)).

The host-produced mucins making up the colonic mucus layer represent a key source of complex carbohydrates within the gut. We demonstrate that the T2SS allows *C. rodentium* to metabolize mucin (Figure S3A,B), a normally unavailable carbon source for commensal bacteria, which can thereby give *C. rodentium* a competitive advantage against the resident microbiota and allow for mucosal population expansion.^60^ Interestingly, the T2SS of *Pseudomonas aeruginosa*, a major causative agent of airway infections, has been shown to be important for lung mucin degradation and its utilization as a carbon source.^61^ Similarly, intestinal pathogen *Vibrio cholerae* uses a type II-secreted GH18 domain-containing effector ChiA2 to directly release β-1, 4 linked N-acetylglucosamine residues (GlcNAc) from mucin, creating catabolically available GlcNAc monomers and oligomers.^52^ Intriguingly, ChiA2 was shown to facilitate degradation of intestinal mucus from several different mammals, indicating a lack of host specificity for this class of enzymes. Therefore mucin degradation may be a conserved function of the T2SS, regardless of pathogen or target body site.

Mucin degradation provides another beneficial outcome for colonization as it allows the pathogen to create a path to intimate attachment to epithelial cells. Indeed, it has been shown that perturbations to the integrity of the intestinal mucus, such as antibiotic treatment in mice, led to increased attachment of *C. rodentium* to intestinal epithelium and exacerbated disease severity.^62^ Similarly, in mice colonized with synthetic human gut microbiota, a depletion of dietary fiber and consequent alteration of the resident microbiota’s consumption patterns toward host-derived mucus glycoproteins, resulted in the thinning of the colonic mucus barrier.^55^ This, in turn, increased susceptibility to lethal colitis caused by *C. rodentium*. While confounding factors such as diet or colonization by mucus-degrading commensal microbes may provide an advantage for invading AE pathogens, pathogens must employ techniques to penetrate the mucus barrier in the absence of these factors. For example, a number of pathogenic *E. coli* strains encode mucinases, such as the metalloprotease YghJ of enterotoxigenic *E. coli* (ETEC) and the EHEC metalloprotease StcE, both of which are associated with facilitating intestinal epithelial cell attachment.^9,63^

Aside from identifying conserved functionality of T2SS substrates across pathogens, of the type II-secreted proteins identified in this study, those encoded at the *ROD_44841–11* locus share homology with several of the secreted effectors found in other pathogens. As previously mentioned, these proteins contain a GH18 domain, which aside from cleaving mucins can also act on other substrates such as chitin and peptidoglycan. One such protein is ChiA of adherent invasive *E. coli* (AIEC), which has been shown to increase adhesion to intestinal epithelial cells via the interaction of its chitin-binding domain (GH18) and the N-glycosylated asparagine of the host factor chitinase-3-like-1.^64^ Another homologous chitinase from *Salmonella* removes the terminal sialic acid moiety from the host cell surface, and facilitates the invasion of the pathogen into the epithelial cells.^65^ As such, it is possible that some of the *C. rodentium* predicted T2SS GH18-containing substrates further facilitate epithelial cell attachment by interacting with host factors distinct from mucin, though further study is required to investigate their activity toward other substrates. Importantly, we demonstrate that secretion of the *ROD_44841–11* effectors is necessary for host colonization, suggesting a similar importance of homologous proteins during human pathogen infection.

Intriguingly, while the loss of the functional T2SS or some of its GH18-containing effectors led to significant colonization defects during infection of the C57BL/6J mice, this phenotype was diminished in a severe model of infection using C3H/HeJ mice. Altered infection dynamics are one of the major contributing factors leading to differences in disease severity between the mouse models. Indeed, profiling of the *C. rodentium* infection in C3H/HeJ and another related susceptible mouse genotype, C3H/HeN, revealed significantly faster and more uniform colonization of the gastrointestinal tract compared to infection in resistant mice.^48,66^ Thus, it is plausible that C3H mice lack some of the colonization resistance mechanisms found in C57BL/6J mice, which allowed the T2SS-deficient strain to colonize at a level comparable to WT *C. rodentium*. Nevertheless, we still observed reduced mortality associated with a loss of the T2SS, suggesting another role of this system aside from aiding colonization (Figure 3(c)). The susceptibility of the C3H mice to fatal infection is largely governed by a robust activation of *R-spondin2* gene, upregulation of the Wnt pathway, and associated loss of epithelial differentiation and increased CD4^+^ T cell activation.^67,68^ Thus, *C. rodentium* T2SS can potentially contribute to some or all of these aspects of severe infection, and the specific effectors and their mechanism of action should be investigated in future studies.

In summary, our study demonstrates that *C. rodentium* possesses a functional T2SS with a vast repertoire of predicted multi-functional effector proteins, primarily utilized for the degradation of general carbohydrates, particularly mucin. Intriguingly, while T2SS are commonly found across pathogens and non-pathogens alike, their expression is usually suppressed in the human-resident nonpathogenic bacteria, such as commensal *E. coli* strains.^5^ This system’s specificity toward pathogens makes it an attractive candidate for therapeutic intervention and a potential alternative to broad-spectrum antibiotics. On the other hand, the increased colonization potential and expanded metabolic repertoire may make the T2SS a useful addition to the engineered probiotic bacteria to ensure successful seeding and establishment in the human intestine.^69^

## Supplementary Material

Citro_T2SS_Supplemental figures_revised.docx

## Data Availability

The authors confirm that the data supporting the findings of this study are available within the article [and/or] its supplementary materials. Raw 16S sequence data from mouse fecal samples have been deposited in the NCBI SRA database with the accession number (PRJNA949387).
