# Supplementary material for: Citrobacter rodentium possesses a functional type II secretion system necessary for successful host infection
Source: Gut Microbes. 2024 Feb 1;16(1):2308049. doi: 10.1080/19490976.2024.2308049 (PMC10841016; doi:10.1080/19490976.2024.2308049)
Supplement: Citro_T2SS_Supplemental figures_revised.docx [file KGMI_A_2308049_SM2774.docx]

Supplementary figures for: “*Citrobacter rodentium* possesses a functional Type II Secretion System necessary for successful host infection”.

Table S1. Strains used in this study

| **Strain name** | **Description/Relevant Genotype or Phenotype** | **Antibiotic Resistance** | **Source** |
| --- | --- | --- | --- |
| *Citrobacter rodentium* DBS100 | Wild-type *C. rodentium* strain |  | Lab stock |
| *C. rodentium* CmR | Wild-type *C. rodentium* strain with chloramphenicol resistance | Chloramphenicol | Lab stock |
| *C. rodentium* Δ*gspK* | *C. rodentium* derivative with a non-polar *gspK* deletion |  | This study |
| *C. rodentium* Δ*gspK CmR* | *C. rodentium* derivative with a non-polar *gspK* deletion with a Tn7 integrated chloramphenicol resistance cassette | Chloramphenicol | This study |
| *C. rodentium* Δ*gspK::gspK* | Chromosomal complementation of Δ*gspK* | Chloramphenicol | This study |
| *C. rodentium* Δ*glpQ* | *C. rodentium* derivative with a non-polar *glpQ* deletion |  | This study |
| *C. rodentium* Δ*ROD_44841-11* | *C. rodentium* derivative with a non-polar *ROD_44841-11* deletion |  | This study |
| *Escherichia coli* MC1061*λpir* | *E. coli* strain for creation of the pRE112-deletion construct |  | Lab stock |
| *E. coli* MFDpir | *E. coli* strain for delivery of the pRE112-deletion construct into *C. rodentium* by conjugation. |  | Lab stock |
| pTNS2 | *E. coli* with a Tn7 transposase expression plasmid | Ampicillin | Choi and Schweizer, 2006 |
| *Citrobacter rodentium* DBS100 + ROD_44811-V5 | Wild-type *C. rodentium* strain carrying a ROD_44811-V5 encoding plasmid | Chloramphenicol | This study |
| *C. rodentium* Δ*gspK + ROD_44811-V5* | *C. rodentium* derivative strain with a non-polar *gspK* deletion carrying a ROD_44811-V5 encoding plasmid | Chloramphenicol | This study |
| *Citrobacter rodentium* DBS100 + ROD_44831-V5 | Wild-type *C. rodentium* strain carrying a ROD_44831-V5 encoding plasmid | Chloramphenicol | This study |
| *C. rodentium* Δ*gspK + ROD_44831-V5* | *C. rodentium* derivative strain with a non-polar *gspK* deletion carrying a ROD_44831-V5 encoding plasmid | Chloramphenicol | This study |
| *Citrobacter rodentium* CmR + TOPO-ROD_44811-V5 | Wild-type C. rodentium strain with chloramphenicol resistance carrying a TOPO-based ROD_44811-V5 encoding plasmid | Kanamycin + Chloramphenicol | This study |
| *C. rodentium* Δ*gspK + TOPO-ROD_44811-V5* | *C. rodentium* derivative with a non-polar *gspK* deletion with a Tn7 integrated chloramphenicol resistance cassette carrying a TOPO-based ROD_44811-V5 encoding plasmid | Kanamycin + Chloramphenicol | This study |
| *C. rodentium* Δ*gspK::gspK + TOPO-ROD_44811-V5* | Chromosomal complementation of Δ*gspK* carrying a TOPO-based ROD_44811-V5 encoding plasmid | Kanamycin + Chloramphenicol | This study |

Table S2. Plasmids used in this study.

| **Plasmid** | **Description** | **Antibiotic Resistance** | **Source** |
| --- | --- | --- | --- |
| pRE112 | Empty suicide vector backbone for bacterial gene knock-out | Chloramphenicol | Edwards *et al.*, 1998 |
| pRE112-gspK | Plasmid for *gspK* knock-out | Chloramphenicol | This study |
| pRE112-glpQ | Plasmid for *glpQ* knock-out | Chloramphenicol | This study |
| pRE112-44841_11 | Plasmid for *ROD_44841-11* knock-out | Chloramphenicol | This study |
| pUC18R6KT-mini-Tn7T | Mini-Tn7 base vector used for constructing chromosomally complemented strains | Ampicillin | Choi and Schweizer, 2006 |
| pTrc99a | Plasmid used as template for cloning trc promoter and C-terminal V5 tag | Ampicillin | Lab stock |
| pBAD33-trc-ROD44831-V5 | Plasmid for IPTG-inducible gene expression of *ROD_44831* C-terminal V5 tag | Chloramphenicol | This study |
| pBAD33-trc-ROD44811-V5 | Plasmid for IPTG-inducible gene expression of *ROD_44811* C-terminal V5 tag | Chloramphenicol | This study |
| TOPO-trc-ROD_44811-V5 | Kanamycin-resistant plasmid for IPTG-inducible gene expression of *ROD_44811* C-terminal V5 tag | Kanamycin | This study |

Table S3. Primers used in this study.

| **Primer** | **Sequence (5'-3')** | **Purpose** |
| --- | --- | --- |
| N-term gspK_fwd | ATTCCCGGGAGAGCT*C*ACTCAGCAAAGCGGCATG | Forward primer to amplify upstream fragment to construct *C. rodentium ΔgspK* mutant |
| N-term gspK_rev | CTGCCGTTAACATATGGATGGTTAGCTCAGC | Reverse primer to amplify upstream fragment to construct *C. rodentium ΔgspK* mutant |
| C-term gspK_fwd | TCCATATGTTAACGGCAGGTGACGAAG | Forward primer to amplify downstream fragment to construct *C. rodentium ΔgspK* mutant |
| C-term gspK_rev | AGCTTCTTCTAGAGGTAC*C*TGGAGGTCAGCTGTTGTTC | Reverse primer to amplify downstream fragment to construct *C. rodentium ΔgspK* mutant |
| n-term glpQ_fwd | CATGAATTCCCGGGAGAGCT*C*GAGCGAATCTTCTCCGGC | Forward primer to amplify upstream fragment to construct *C. rodentium ΔglpQ* mutant |
| n-term glpQ_rev | CGGCATGGTGGAGAAATAAACCGTTAGCGAAAG | Reverse primer to amplify upstream fragment to construct *C. rodentium ΔglpQ* mutant |
| C-term-glpQ_fwd | TTTATTTCTCCACCATGCCGGCCATCATC | Forward primer to amplify downstream fragment to construct *C. rodentium ΔglpQ* mutant |
| C-term-glpQ_rev | CAAGCTTCTTCTAGAGGTAC*C*ACTGGTGGTCGCAGAAGG | Reverse primer to amplify downstream fragment to construct *C. rodentium ΔglpQ* mutant |
| n-term ROD_44841-11_fwd | CATGAATTCCCGGGAGAGCT*C*TGGTTGAGATGGAAGTGCGTG | Forward primer to amplify upstream fragment to construct *C. rodentium ΔROD_44841-11* mutant |
| n-term ROD_44841-11_rev | TATTTGATTCAGCAAATGCCGGCAACGC | Reverse primer to amplify upstream fragment to construct *C. rodentium ΔROD_44841-11* mutant |
| C-term-ROD_44841-11_fwd | GGCATTTGCTGAATCAAATATGCCAGGATATAATCC | Forward primer to amplify downstream fragment to construct *C. rodentium ΔROD_44841-11* mutant |
| C-term-ROD_44841-11_rev | CAAGCTTCTTCTAGAGGTAC*C*CGCCGAATTTCCGTGATTC | Reverse primer to amplify downstream fragment to construct *C. rodentium ΔROD_44841-11* mutant |
| pBAD33-map-V5_fwd | TTTTTATCCATAAGATTAGCGAGCTGTTGACAATTAATCATCCG | Forward primer to amplify trc-effector-V5 sequence from pTrc99a plasmid and insert into pBAD33 |
| pBAD33-map-V5_rev | TGCATGCCTGCAGGTCGACT*CTAGA*TTAGGTGCTGTCCAGGCC | Reverse primer to amplify trc-effector-V5 sequence from pTrc99a plasmid and insert into pBAD33 |
| ROD_44811_fwd | ATGGAATTCGAGCTCGGTACCATGAAATTAAATTTAGCCACTAAG | Forward primer to amplify *ROD_44811* and clone it into pBAD33-trc-V5 plasmid digested with BamHI and KpnI |
| ROD_44811_rev | GTTGGGGATGGGCTTGCCTGGATCCTTTTTAAGCATATCTTCCGTC | Reverse primer to amplify *ROD_44811* and clone it into pBAD33-trc-V5 plasmid digested with BamHI and KpnI |
| ROD_44831_fwd | ATGGAATTCGAGCTCGGTACCATGAAGCTCAATAAATTAACTTC | Forward primer to amplify *ROD_44831* and clone it into pBAD33-trc-V5 plasmid digested with BamHI and KpnI |
| ROD_44831_rev | GTTGGGGATGGGCTTGCCTGGATCCCTTCAACATATCTTCGGTAAG | Reverse primer to amplify *ROD_44841* and clone it into pBAD33-trc-V5 plasmid digested with BamHI and KpnI |
| gspA-Citro-f | TGCTTAACCTTGAGGCGGAG | Forward primer for qPCR assessment of expression of *gspA* |
| gspA-Citro-r | AGAGAAAGCTGCCCCATGAC | Forward primer for qPCR assessment of expression of *gspA* |
| gspC-Citro-f | GATCACCGCCATAAGCCGTA | Forward primer for qPCR assessment of expression of *gspC* |
| gspC-Citro-r | ATGTAATCCGCCAGCGCTAA | Forward primer for qPCR assessment of expression of *gspC* |
| gspD-Citro-f | GCGACACACCTTGATACCCA | Forward primer for qPCR assessment of expression of *gspD* |
| gspD-Citro-r | CTGCGGTAATGGAAATGGCG | Forward primer for qPCR assessment of expression of *gspD* |
| gspE-Citro-f | CGCAATATTGACGTTCGCGT | Forward primer for qPCR assessment of expression of *gspE* |
| gspE-Citro-r | GTTGGTCCGGTGACCAGAAT | Forward primer for qPCR assessment of expression of *gspE* |
| q-44841-f | GATTTGCAGAAAGAAGGGAAAG | Forward primer for qPCR assessment of expression of *ROD_44841* |
| q-44841-r | GTGAGCTGGCTGAAGTTAAT | Forward primer for qPCR assessment of expression of *ROD_44841* |
| q-44831-f | CCCAGGAAGGCATCAATAAA | Forward primer for qPCR assessment of expression of *ROD_44831* |
| q-44831-r | CTGGTGAAGTTTGCCCTT | Forward primer for qPCR assessment of expression of *ROD_44831* |
| q-44821-f | GTTGCTTCAGGAACCGATAG | Forward primer for qPCR assessment of expression of *ROD_44821* |
| q-44821-r | CCATTGCTGGTTGATTGTAAAG | Forward primer for qPCR assessment of expression of *ROD_44821* |
| q-44811-f | AAAGTCGGTACGCCTAAATC | Forward primer for qPCR assessment of expression of *ROD_44811* |
| q-44811-r | GCCATAGTGAAGTGGGTTAC | Forward primer for qPCR assessment of expression of *ROD_44811* |
| q-glpQ-f | CGGCCAGTAATACGGATAAAG | Forward primer for qPCR assessment of expression of *glpQ* |
| q-glpQ-r | CCAGATCCTGCTCCAGATA | Forward primer for qPCR assessment of expression of glpQ |
| q-dnaQ-f | GTCAGGCCCGCAAATTAC | Forward primer for qPCR assessment of expression of *dnaQ* |
| q-dnaQ-r | TCTCCACCAGATCGAGACG | Forward primer for qPCR assessment of expression of *dnaQ* |
| 515F | GTGCCAGCMGCCGCGGTAA | Forward primer to amplify 16S rRNA V4 |
| 806R | GGACTACHVHHHTWTCTAAT | Reverse primer to amplify 16S rRNA V4 |
| PTn7R | CACAGCATAACTGGACTGATTTC | Forward primer for confirming chromosomal insertion of mini-Tn7 elements |
| PglmS-down_Citro | GCACGTTGAGGAAGTCATTGC | Reverse primer for confirming chromosomal insertion of mini-Tn7 elements |
| Tn7-check_FW | tagttgggaactgggagggg | Forward primer for confirming fragment insertion in mini-Tn7 |
| Tn7-check_RV | tccgaagttcctattctctagaaagt | Reverse primer for confirming fragment insertion in mini-Tn7 |
| EP.gspK_1.F | ggcctgcaaggccttcgcgaggtaccATCAGTAAAATTAATAATATTTTCTTAGCGAATTTTAAAGCCTAATT | Set of primers to amplify *gspK* along with its native promoter for in-chromosomal complementation |
| EP.gspK_1.R | GCATTGTTCTTCTCCGGGGTATAATTAATAAATAGCGATAAGATTCCTCTTTTTTTAAAAAAAAGCAA |  |
| EP.gspK_2.F | ATCGCTATTTATTAATTATACCCCGGAGAAGAACAATGCAAAATGAG |  |
| EP.gspK_2.R | ggatcccccgggctgcaggaattcctcgagTTATTCTTCGTCACCTGCCGTTAACTGG |  |


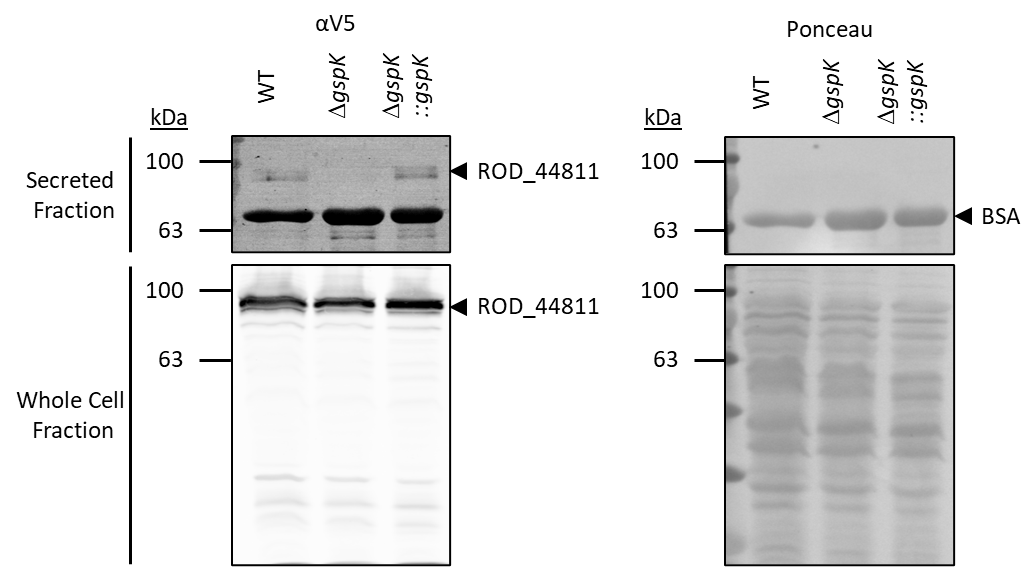


Supplemental Figure S1. Protein secretion assay showing complementation of secretion of putative effector protein ROD_44811 in the isogenic chloramphenicol-resistant WT, ∆*gspK* and ∆*gspK::gspK C. rodentium* strains. Western blot detection of the ectopically expressed C-terminally V5-tagged ROD_44811 protein. Ponceau staining for BSA (secreted fraction), or total protein (whole cell fraction) shown to demonstrate equal loading.


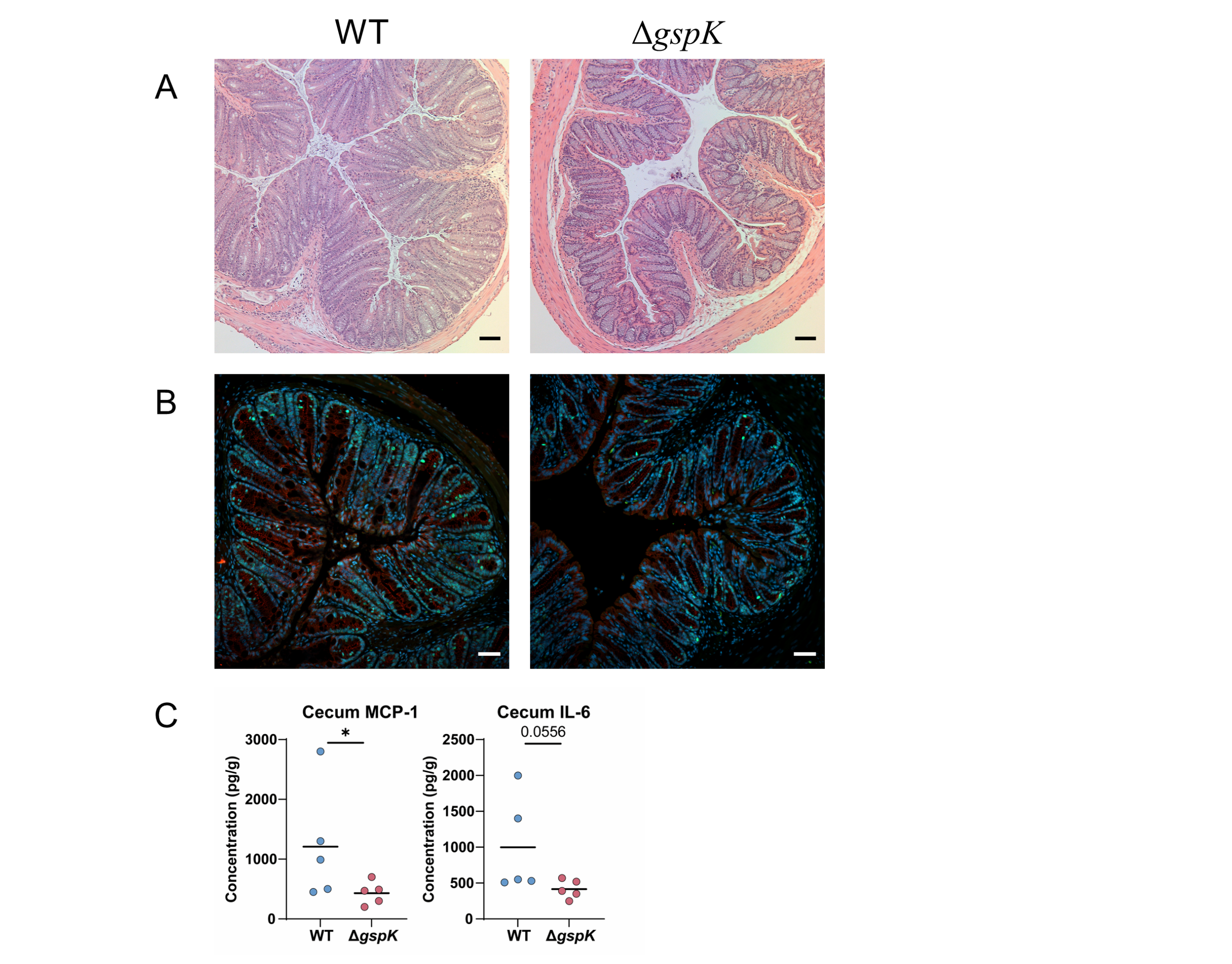


Supplemental Figure S2. *C. rodentium* T2SS is necessary for successful infection in C57BL/6 mice. (A) Representative images of the H&E-stained C57BL/6J mouse colon infected with either WT (left) or Δ*gspK* (right) *C. rodentium.* Scale bar = 50 µm. (B) Representative immunofluorescence images of the C57BL/6J mouse colon infected with either WT (left) or Δ*gspK* (right) *C. rodentium.* CDH1 staining is in red, Ki67 staining is in green, and DAPI is in blue. Scale bar = 50 µm. (C) Levels of proinflammatory cytokines MCP-1 and IL-6 in the cecum were assessed with a cytometric bead assay (CBA). n = 5, * denotes *p* < 0.05 measured by Wilcoxon signed-rand rank test.


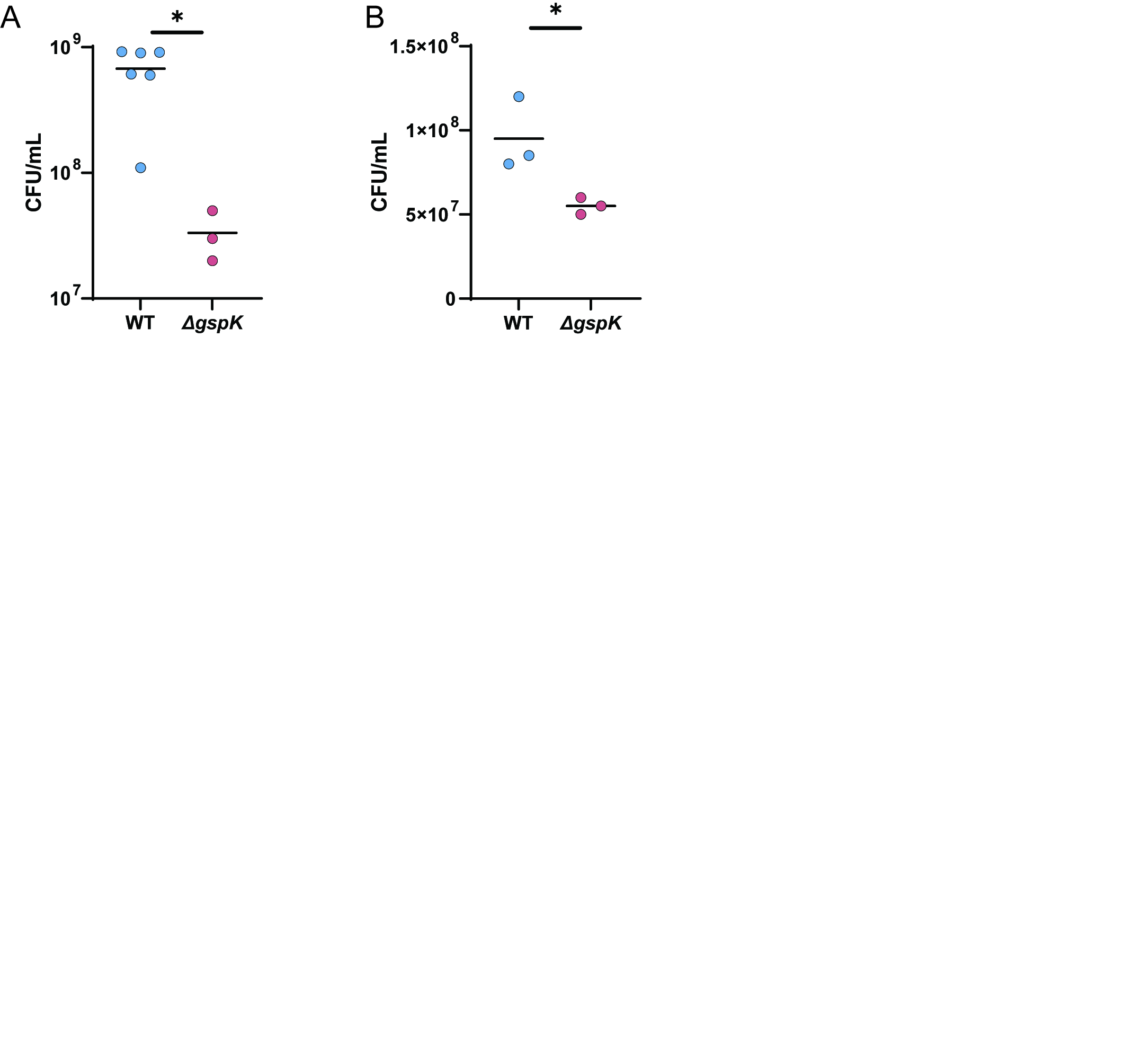


Supplemental Figure S3. T2SS supports *C. rodentium* growth on mucin. (A) Bacterial load of WT and ∆*gspK* *C. rodentium* in mucin-rich media following 24 hours of growth. * denotes *p* < 0.05 measured by Wilcoxon signed-rand rank test. (B) Bacterial load of WT and ∆*gspK C. rodetium* in minimal media supplemented with 1% mucin as a sole carbon source following 24 hours of growth. * denotes *p* < 0.05 measured by Wilcoxon signed-rand rank test.


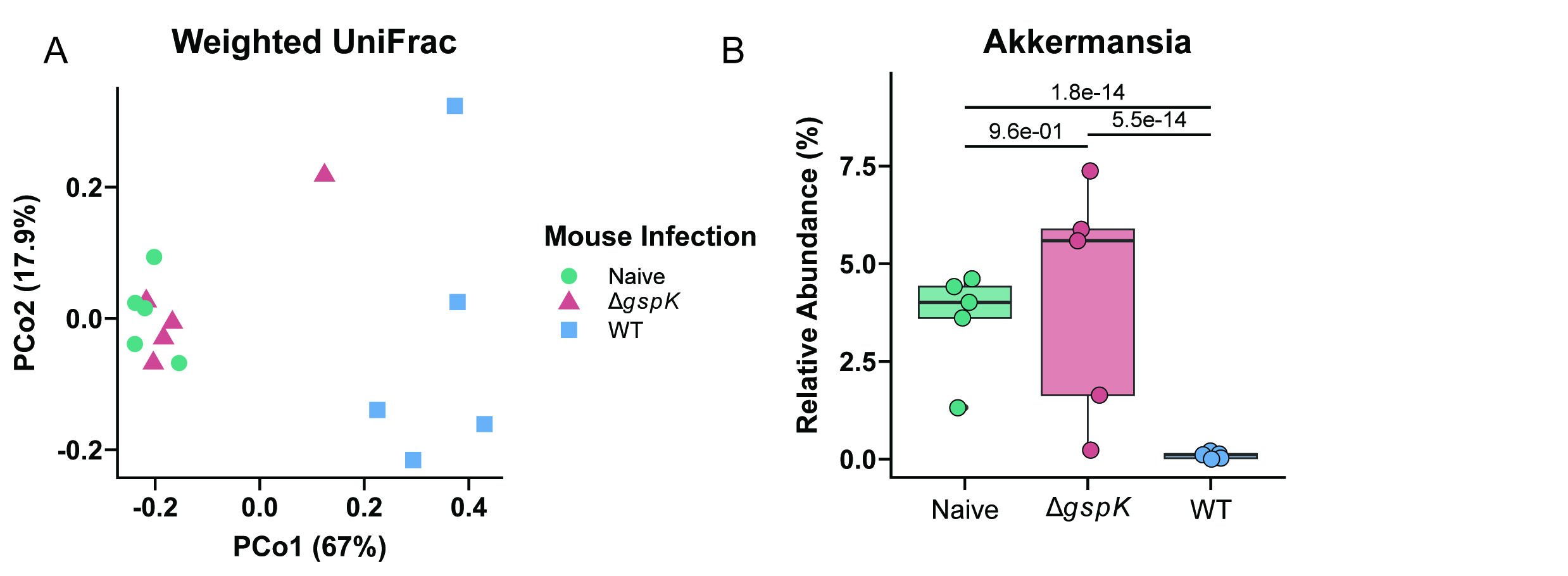


Supplemental Figure S4. T2SS is necessary for resident microbiota displacement during infection. (A) Principal coordinate analysis of the microbiota of naïve mice, and those infected with WT or T2SS-deficient (Δ*gspK*) C. rodentium. Each point represents an individual mouse. Samples with similar microbial communities are clustered together. n = 5 for each group. (B) Relative abundance of the genus *Akkermansia*. *p*-values represent the adjusted *p*-values from DESeq2 analysis.


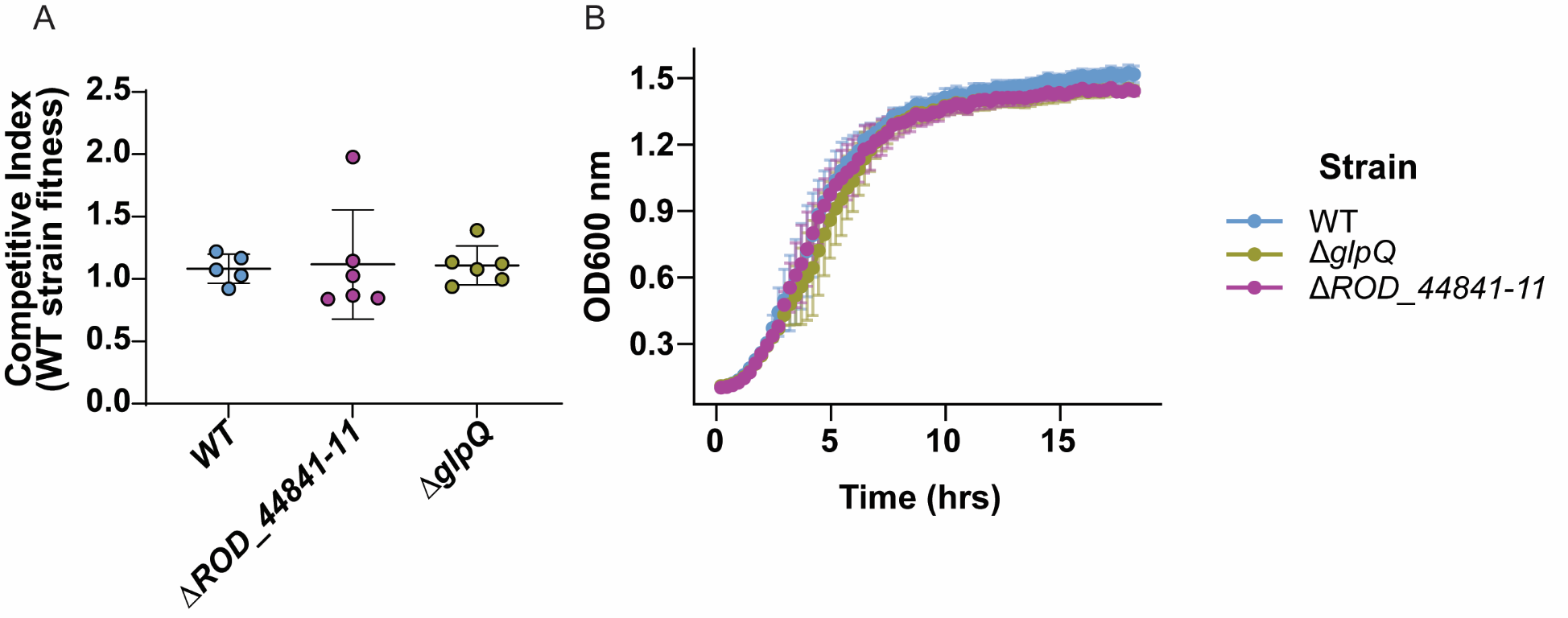


Supplemental Figure S5. Individual T2SS effector genes are not required for epithelial cell attachment *in vitro*. (A) Competitive index (CI) calculated via co-infection of intestinal epithelial cell line HT-29-MTX with either WT, Δ*ROD_44841-11,* or Δ*glpQ* *C. rodentium* strains and chloramphenicol-resistant isogenic wildtype *C. rodentium*. CI is indicative of the proportion of adherent bacteria belonging to each strain following a 4-hour infection period compared to the corresponding ratio of strains in the inoculating population. Kruskal-Wallis test followed by Dunn’s multiple comparisons test. n = 5-6. (B) Putative T2SS effector deletion did not affect bacterial growth in vitro. Growth dynamics of WT or effector deletion strains in LB measured by optical density of the culture. n = 3, error bars are standard deviation.


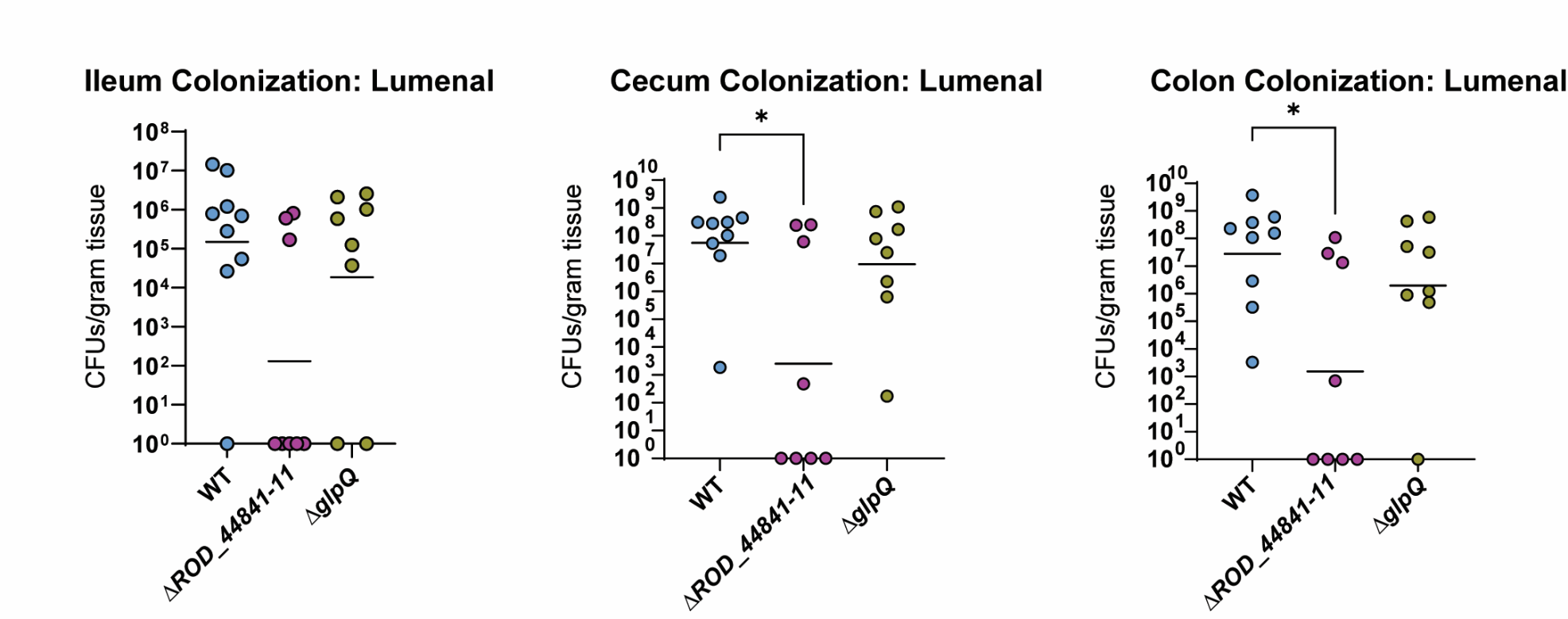


Supplemental Figure S6. Individual T2SS effectors aid successful lumenal colonization in C57BL/6 mice. Lumenal sub-populations of *C. rodentium* in cecum, ileum, and colon were measured by plating the lumenal contents on MacConkey agar and counting CFU. Wilcoxon signed-rank test. n = 5-10, * - *p* < 0.05.
